# Effects of *p*-Cresol on Oxidative Stress, Glutathione Depletion, and Necrosis in HepaRG Cells: Comparisons to Other Uremic Toxins and the Role of *p*-Cresol Glucuronide Formation

**DOI:** 10.3390/pharmaceutics13060857

**Published:** 2021-06-09

**Authors:** Sang Zhu, Yan Rong, Tony K. L. Kiang

**Affiliations:** Katz Group Centre for Pharmacy and Health Research, Faculty of Pharmacy and Pharmaceutical Sciences, University of Alberta, Edmonton, AB T6G 2E1, Canada; sang1@ualberta.ca (S.Z.); yrong1@ualberta.ca (Y.R.)

**Keywords:** *p*-cresol, *p*-cresol glucuronide, *p*-cresol sulfate, uremic toxins, HepaRG, metabolism

## Abstract

The toxicological effects of *p*-cresol have primarily been attributed to its metabolism products; however, very little human data are available in the key organ (i.e., liver) responsible for the generation of these metabolites. Experiments were conducted in HepaRG cells utilizing the following markers of cellular toxicity: 2′-7′-dichlorofluorescein (DCF; oxidative stress) formation, total cellular glutathione (GSH) concentration, and lactate dehydrogenase (LDH; cellular necrosis) release. Concentrations of *p*-cresol, *p*-cresol sulfate, and *p*-cresol glucuronide were determined using validated assays. *p*-Cresol exposure resulted in concentration- and time-dependent changes in DCF (EC_50_ = 0.64 ± 0.37 mM at 24 h of exposure) formation, GSH (EC_50_ = 1.00 ± 0.07 mM) concentration, and LDH (EC_50_ = 0.85 ± 0.14 mM) release at toxicologically relevant conditions. *p*-Cresol was also relatively more toxic than 3-carboxy-4-methyl-5-propyl-2-furanpropanoic acid, indole-3-acetic acid, indoxyl sulfate, kynurenic acid, and hippuric acid on all markers. Although the exogenous administration of *p*-cresol sulfate and *p*-cresol glucuronide generated high intracellular concentrations of these metabolites, both metabolites were less toxic compared to *p*-cresol at equal-molar conditions. Moreover, *p*-cresol glucuronide was the predominant metabolite generated in situ from *p*-cresol exposure. Selective attenuation of glucuronidation (without affecting *p*-cresol sulfate formation, while increasing *p*-cresol accumulation) using independent chemical inhibitors (i.e., 0.75 mM l-borneol, 75 µM amentoflavone, or 100 µM diclofenac) consistently resulted in further increases in LDH release associated with *p*-cresol exposure (by 28.3 ± 5.3%, 30.0 ± 8.2% or 27.3 ± 6.8%, respectively, compared to *p*-cresol treatment). These novel data indicated that *p*-cresol was a relatively potent toxicant, and that glucuronidation was unlikely to be associated with the manifestation of its toxic effects in HepaRG cells.

## 1. Introduction

*p*-Cresol, a part of the protein-bound uremic toxin milieu, is derived from colonic amino acids tyrosine and phenylalanine [1]. The toxicological effects of *p*-cresol have primarily been attributed to its metabolism end products [2], which are considered relatively significant toxic species amongst a large variety of uremic toxins known to date [2,3]. *p*-cresol is extensively conjugated in enterocytes and hepatocytes in the formation *p*-cresol sulfate and *p*-cresol glucuronide [1,4]. Under typical uremic conditions, sulfonation is the predominant pathway, evidenced by the relatively higher plasma total concentrations of *p*-cresol sulfate in various clinical reports [5,6,7,8,9,10]. However, due to differences in protein binding, the biologically active unbound concentrations of *p*-cresol glucuronide and *p*-cresol sulfate are comparable [6,7,8,9], and higher plasma concentrations of the free glucuronide have been documented [10]. Furthermore, a shift to the production of *p*-cresol glucuronide from *p*-cresol sulfate has been observed in patients with advanced kidney disease [5,7], indicating glucuronidation might be an important pathway for *p*-cresol metabolism at higher *p*-cresol concentrations where the toxicity is more likely manifested.

*p*-Cresol glucuronide has been implicated in cardiovascular toxicity and overall mortality in human models. Liabeuf et al. [6] were the first to establish an association between serum *p*-cresol glucuronide concentrations and total or cardiac-related mortality, highlighting similar predictive powers of the glucuronide compared to the sulfate metabolite. Consistent observations were also reported by Glorieux et al. [3] in chronic kidney disease patients not yet on hemodialysis, where both plasma concentrations of total and unbound *p*-cresol glucuronide were correlated with total mortality. In patients with advanced chronic kidney disease, the plasma ratio of *p*-cresol glucuronide to *p*-cresol sulfate is progressively increased [5,7], and this apparent shift in metabolic profile from sulfonation to glucuronidation has also been associated with cardiac disease and mortality [5]. A role of *p*-cresol glucuronide in early stages of cardiac disease progression has also been suggested in chronic kidney disease patients without full-blown cardiac dysfunction, where both free and total serum *p*-cresol glucuronide concentrations have been associated with decreased peak cardiac power, peak cardiac output, mean arterial pressure, peak heart rate, and aerobic exercise capacity [9]. Furthermore, experimental data supported the nephrotoxic effects of the glucuronide metabolite. Exogenously administered *p*-cresol glucuronide (2 mM) generated mitochondrial toxicity [11] and altered gene expressions associated with epithelial-to-mesenchymal transition [7] in conditionally immortalized human renal proximal tubule epithelial cells. In human embryonic kidney 293 cells, exposure of 100 µM of *p*-cresol glucuronide for seven days also resulted in modest reductions in cell viability [12]. In addition, toxicity data of *p*-cresol glucuronide are also available in other human tissues. In an ex vivo model, leukocytes isolated from the whole blood of healthy subjects exposed to 48 mg/L of *p*-cresol glucuronide for 10 min further enhanced the oxidative burst activities associated with *p*-cresol sulfate [10]. In primary cultures of human hepatocytes, the viability, cellular ATP concentration, and mitochondrial membrane potential were significantly reduced in cells exposed to 0.5 mM of *p*-cresol glucuronide for 96 h [13].

While these data collectively suggested the potential toxicity profiles of *p*-cresol glucuronide, the clinical studies were primarily correlational in nature, and most in vitro/ex vivo experimental models have utilized exogenously administered *p*-cresol glucuronide at relatively high concentrations. Very few studies have also provided direct comparisons on the toxic effects of *p*-cresol (i.e., pre-cursor) to its metabolites, where the limited available data appeared to suggest that *p*-cresol was equally, if not more toxic, than its glucuronide [11,12]. Furthermore, despite evidence supporting the toxicity of *p*-cresol glucuronide and *p*-cresol sulfate, very little human data are available in the key organ (i.e., liver) responsible for the generation of these metabolites. Although *p*-cresol-induced liver injury has already been documented in several pre-clinical animal studies [14,15,16,17], the relative toxic effects of *p*-cresol (which is more abundant than its conjugated metabolites in the human liver [18]) and the role of in situ-generated metabolites in the manifestation of toxicity in a human experimental hepatic model have not yet been systematically characterized.

To further elucidate the toxicological importance of *p*-cresol in a liver model and determine whether the glucuronidation of *p*-cresol constituted a toxification or a detoxification pathway, our objectives were to (i) systematically characterize the effects of *p*-cresol in comparison to other protein-bound uremic toxins and its metabolites on markers of oxidative stress (2′-7′-dichlorofluorescein, DCF, formation), total cellular glutathione (GSH) concentration, and cellular necrosis (lactate dehydrogenase, LDH, release), which are toxicity endpoints known to be associated with *p*-cresol or its metabolites in various other liver experimental models (e.g., [13,16,17,19]); (ii) determine the metabolic profiles of *p*-cresol in the generation of its conjugated metabolites; and (iii) characterize the role of in situ-generated *p*-cresol glucuronide in the manifestation of toxicities. We utilized a human primary hepatoma liver cell line (i.e., HepaRG) which is metabolically competent (i.e., allowing the in situ generation and the mechanistic modulation of *p*-cresol metabolites) and known to be suitable for toxicity studies [20,21,22].

## 2. Materials and Methods

### 2.1. Chemicals and Reagents

Ammonium acetate (catalogue# 1220-1-70) was obtained from Caledon Laboratories Ltd. (Georgetown, ON, Canada) and further passed through Millipore™ Millex™ 0.45 µM filters from Fisher Scientific (Mississauga, ON, Canada) before being added to mobile phases. Glutathione assay kit (catalogue# 703002) was purchased from Cayman Chemical Company (Ann Arbor, MI, USA). Amentoflavone (catalogue# 40584), 3-carboxy-4-methyl-5-propyl-2-furanpropionic acid (CMPF, catalogue# 90833), 2′, 7′-dichlorofluorescin diacetate (DCFDA, catalogue# D6883), diclofenac (catalogue# D6899), 2,6-dimethylphenol (DMP, catalogue# D174904), formic acid (catalogue# F0507), hydrocortisone 21-hemisuccinate sodium salt (catalogue# H2270), hippuric acid (catalogue# 112003), indole-3-acetic acid (catalogue# I2886), indoxyl sulfate potassium salt (catalogue# I3875), kynurenic acid (catalogue# K3375), lactate dehydrogenase cytotoxicity detection kit (catalogue# 4744926001), l-borneol (catalogue# 15598), meta-phosphoric acid (catalogue# 239275), methanol (catalogue# 34860), tert-butyl hydroperoxide (t-BOOH, catalogue# 416665), triethanolamine (catalogue# T58300), Triton X-100 (catalogue# T8787), *p*-cresol (catalogue# C85751), penicillin-streptomycin (catalogue# P0781), and water (catalogue# 270733) were purchased from Sigma-Aldrich (Oakville, ON, Canada). *p*-Cresol glucuronide (catalogue# C782005), *p*-cresol glucuronide-d_7_ (catalogue# C782007), *p*-cresol sulfate potassium salt (catalogue# T536805), and *p*-cresol sulfate potassium salt-d_7_ (catalogue# T536802) were purchased from Toronto Research Chemicals (North York, ON, Canada). Dimethyl sulfoxide (DMSO, catalogue# D2650), Dulbecco’s phosphate-buffered saline (D-PBS, catalogue# 14190-144), fetal bovine serum (FBS, catalogue# 12483-020), human recombinant insulin (catalogue# 12585-014), L-glutamine (GlutaMax, catalogue# 35050-061), trypsin (0.05%)/ethylenediaminetetraacetic acid (EDTA, catalogue# 25300-062), and Williams’ E medium (catalogue# 12551-032) were purchased from Thermo Fisher Scientific (Ottawa,, ON, Canada). Sodium EDTA (catalogue# CH110) was purchased from Truin Science (Edmonton, AL, Canada).

### 2.2. HepaRG Cell Maintenance and Differentiation

HepaRG is a hepatic tumor cell line that was established from a female subject infected with hepatitis C virus [21]. The HepaRG cell line exhibits comparable metabolic activities to primary human hepatocytes, making it a suitable tool for metabolism and xenobiotic toxicity studies [20,21,22]. HepaRG cells were obtained from Biopredic International (Rennes, France) via a material transfer agreement with Inserm Transfert (Paris, France). Cells were maintained and differentiated according to Biopredic International and Gripon et al.’s protocols [21,23,24]. William’s E medium was supplemented with 10% FBS, 100 units/mL penicillin, 100 µg/mL streptomycin, 5 μg/mL insulin, 5 × 10^−5^ M hydrocortisone hemisuccinate, and 1% glutamine to obtain the growth medium. HepaRG cells were cultured at a density of 0.5 × 10^6^ cells per 25 cm^2^ flasks (Corning, NY, USA) with the growth medium refreshed every 72 h. Cells were passaged every 2 weeks until being subjected to differentiation using the differentiation medium (i.e., growth medium with 2% DMSO, *v*/*v*). After plating onto 24-well culture plates (BioLite, Thermo Fisher Scientific, Ottawa, ON, Canada) using a density of 0.4 × 10^6^ cells/well in 500 μL of differentiation medium, cells were further acclimatized for 12 days before treatment. For all of these procedures, cells were maintained in a 37 °C incubator with 5% CO_2_ (Steri-Cycle CO_2_ incubator, Thermo Fisher Scientific, Mississauga ON, Canada).

### 2.3. HepaRG Cell Treatment

**Figure 1:**
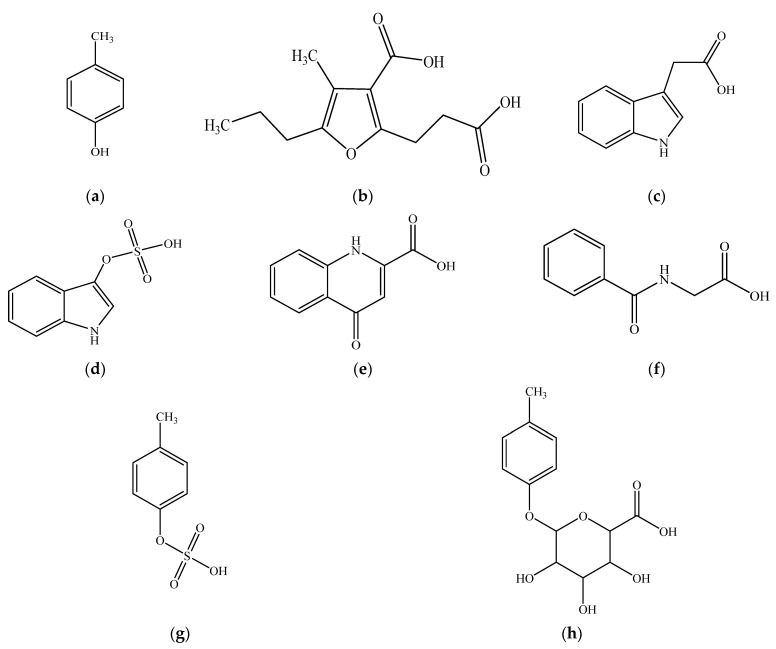
Chemical structures of (**a**) *p*-cresol (CAS# 106-44-5), (**b**) CMPF (CAS# 86879-39-2), (**c**) indole-3-acetic acid (CAS# 87-54-1), (**d**) indoxyl sulfate (CAS# 487-94-5), (**e**) kynurenic acid (CAS# 492-27-3), (**f**) hippuric acid (CAS# 495-69-2), (**g**) *p*-cresol sulfate (CAS# 3233-58-7), and (**h**) *p*-cresol glucuronide (CAS# 17680-99-8). CMPF, 3-carboxy-4-methyl-5-propyl-2-furanpropanoic acid.

All experiments were conducted in differentiated HepaRG cells at passage #18 for consistency. All chemicals described below were soluble in the differentiation medium. Initial control experiments exposed HepaRG cells to a known hepatotoxicant in various liver cellular models, t-BOOH (specific conditions presented below) (e.g., [25,26,27]), to verify the responsiveness of the HepaRG model. To characterize the cellular responses to *p*-cresol, concentration- and time-dependent effects were initially characterized. The stock solution of *p*-cresol (100 mM) was freshly prepared before each treatment and further diluted to treatment concentrations in the differentiation medium. For concentration-response experiments, cells were treated with 0 to 2 mM of *p*-cresol for 24 h. For time-course experiments, cells were treated with 0.75 mM or 1 mM of *p*-cresol for 0 to 24 h (concentrations selected based on linearity in concentration-response data). As *p*-cresol has not been comparatively investigated against other uremic toxins in a hepatic model, an initial experiment determined the toxic effects of *p*-cresol in relation to other protein-bound uremic solutes deemed toxicologically relevant [2,28] (chemical structures shown in Figure 1) to assess the suitability of a focused investigation on *p*-cresol alone. Cells were treated with the differentiation medium (i.e., the vehicle control), 1 mM each of *p*-cresol, 3-carboxy-4-methyl-5-propyl-2-furanpropanoic acid, indole-3-acetic acid, indoxyl sulfate, kynurenic acid, or hippuric acid for 24 h. The effects of these treatments on markers of cellular toxicity (i.e., DCF, GSH, and LDH assays) and *p*-cresol metabolite formations were determined (please see individual toxicity markers and metabolite characterizations below).

To determine the relative effects of exogenously administered *p*-cresol metabolites (Figure 1), cells were exposed to equal-molar concentrations (i.e., 1 mM) of *p*-cresol, *p*-cresol sulfate, and *p*-cresol glucuronide for 24 h prior to toxicity and metabolite assay characterizations. To determine the effects of in situ-generated *p*-cresol glucuronide in the manifestation of cellular toxicity, chemical inhibitors were utilized to selectively attenuate its formation in HepaRG cells. Control experiments were conducted to optimize the specificity (toward *p*-cresol glucuronide formation), potency, and toxicity of several chemical inhibitors: HepaRG cells were pre-treated with blank cell differentiation medium (i.e., the vehicle control), or several concentrations of l-borneol, amentoflavone, or diclofenac for 30 min; then, each inhibitor was co-treated with blank cell differentiation medium or 0.75 mM of *p*-cresol for 24 h. These chemical inhibitors were selected based on known depletion effects of uridine 5′-diphosphoglucuronic acid (UDPGA) (i.e., l-borneol) [29] or inhibitory effects toward UD*P*-glucuronosyltransferase (UGT)1A6 (i.e., amentoflavone and diclofenac) [30,31,32], the latter being the primary enzyme responsible for the production of *p*-cresol glucuronide in human liver microsomes [32]. The optimized inhibitor concentrations (i.e., 0.75 mM l-borneol, 75 µM amentoflavone, and 100 µM diclofenac, to be discussed further in Section 3) that did not generate cellular toxicity (by LDH assay) were utilized in subsequent modulation experiments. *p*-Cresol at 0.75 mM was selected for inhibition experiments based on linear responses in the individual toxicity assays observed in concentration and time-course experiments. The effects of these treatments on markers of cellular toxicities (i.e., DCF, GSH, and LDH assays) were characterized. Concentrations of *p*-cresol, *p*-cresol sulfate, and *p*-cresol glucuronide in culture were also measured as part of control experiments.

### 2.4. DCF Assay for the Measurement of Cellular Oxidative Stress

The formation of the fluorescent DCF from 2′,7′-dichlorofluorescin diacetate (DCFDA) is considered a non-specific marker reaction representative of cellular oxidative stress [33]. Following treatment (as described above in Section 2.3. HepaRG cell treatment), cells were washed twice with 500 μL D-PBS (room temperature [i.e., 23.5 °C]) and exposed to 2 μM DCFDA in 500 μL D-PBS [26]. The fluorescence intensity of the DCF product was measured at excitation and emission wavelengths of 485 nm and 530 nm, respectively, every 3 min up to 30 min to ensure the linearity of DCF formation (SpectraMax M2, Molecular Devices, San Jose, CA, USA). This process was shielded from laboratory incandescent lighting to minimize artefactual DCF formation [33]. DCF formation in treated cells was expressed as a percentage of the vehicle control, after subtracting background fluorescence intensity at 0 min of incubation (which exhibited comparable DCF fluoresce values as cell-free medium). t-BOOH (5 mM, exposed for 10 min to HepaRG cells) was used as the positive control for DCF formation.

### 2.5. GSH Assay for the Measurement of Total Cellular Glutathione Concentration

Total cellular GSH concentration was determined using the manufacturer’s protocol [34] as described by Kiang et al. [27]. Following cell treatment (as described above in Section 2.3. HepaRG cell treatment), cells were washed twice with 500 μL D-PBS (25 °C) and homogenized in 250 μL cold 50 mM 2-(*N*-morpholino) ethanesulphonic acid (MES) buffer containing 0.25 mM EDTA. The resulting cell suspension was sonicated (75D ultrasonic cleaner, VWR International, Mississauga, AL, Canada) for 5 min in ice water, vortexed (Vortex-Genie 2, Thermo Fisher Scientific) for 5 s, and centrifuged (centrifuge 5424 R, Eppendorf, Mississauga, ON, Canada) at 10,000× *g* for 15 min at 4 °C. Subsequently, 200 μL of the supernatant was de-proteinated using 200 μL of meta-phosphoric acid (100 mg/mL) for 5 min and centrifuged at 2300× *g* for 3 min at room temperature (i.e., 23.5 °C). The resulting protein-free supernatant (200 μL) was neutralized with 10 μL of 4 M triethanolamine and incubated (50 μL/well in 96-well plate) with 150 µL of assay reaction mixture on an orbital shaker (VWR Standard Orbital Shaker 3500, Edmonton, AL, Canada) shielded from light at room temperature for 5 min. The assay reaction mixture consisted of 5,5′-dithiobis-2-nitrobenzoic acid, NADP^+^, glucose-6-phosphate, glutathione reductase, glucose-6-phosphate dehydrogenase, and water in MES buffer [34]. The blank control was 50 mM MES buffer. The absorbance of the reaction product, 5-thio-2-nitrobenzoic acid, was measured at 412 nm [34] every 3 min up to 30 min to ensure the linearity of product formation (SpectraMax M2 plate reader, Molecular Devices, San Jose, CA, USA). GSH concentration was calculated using a standard curve ranging from 0–12.5 μM prepared from glutathione disulfide analytical standards [34]. Total cellular GSH concentration in treated cells were expressed as percentages of the vehicle control. t-BOOH (5 mM, exposed for 24 h to HepaRG cells) was used as the positive control for total cellular glutathione depletion.

### 2.6. LDH Assay for the Measurement of Cellular Necrosis

Cellular LDH release as a marker of necrosis was determined using the manufacture’s protocol [35] as described previously [23,26]. Following cell treatment (as described above in Section 2.3. HepaRG cell treatment), cell supernatant was collected and stored on ice. Cells were incubated in 500 µL lysis buffer (i.e., cell differentiation medium with 2% *v/v* Triton X-100 and 20 mM EDTA) for 5 min at room temperature (i.e., 23.5 °C) to facilitate cell detachment. The resulting cell suspension was vortexed for 30 s and centrifuged at 20,000× *g* at 4 °C for 10 min to release cellular LDH. Subsequently, 50 µL each of cell supernatant or processed cell lysates were incubated with 100 µL of the assay reaction mixture (i.e., 200 μL catalyst (i.e., NAD^+^ and diaphorase) and 9000 μL dye solution (i.e., iodotetrazolium chloride and sodium lactate)) in 96-well plates to start the enzyme reaction. The differentiation medium was used as the blank control of which absorbance was subtracted from all measurements. The absorbance of the reaction product, a red formazan salt, was measured at a wavelength of 490 nm [35] every 3 min for up to 30 min to ensure linear enzymatic conditions (SpectraMax M2, Molecular Devices, San Jose, CA, USA). LDH in the cell supernatant was expressed as a percentage of the sum total of LDH quantified from both supernatant and cell lysates [26]. t-BOOH (50 mM, exposed for 24 h to HepaRG cells) was used as the positive control for cellular necrosis.

### 2.7. Quantification of p-Cresol Sulfate and p-Cresol Glucuronide Concentrations in HepaRG Cell Culture

An ultra-high performance liquid chromatography-tandem mass spectrometry assay (UPLC-MS/MS, Shimadzu LC-MS 8050, Kyoto, Japan) was developed and validated in our lab to quantify the concentrations of *p*-cresol sulfate and *p*-cresol glucuronide in HepaRG cell culture, based on a previously published assay [32]. Briefly, culture supernatant (30 µL) or lysates (obtained after removing supernatant, washing cells twice with 500 µL of 37 °C D-PBS, and harvesting cells with 500 µL of 37 °C culture medium) was collected and deproteinated with a mixture of 1 µg/mL *p*-cresol sulfate-d_7_ and *p*-cresol glucuronide-d_7_ (i.e., internal standards) in 90 µL methanol (i.e., extraction solvent) [36]. Analyte extraction was shielded from light at room temperature (i.e., 23.5 °C) for 20 min. In order to precipitate proteins, the mixture was vortexed, sonicated (each for 30 s), and centrifuged at 4000× *g* for 10 min at 4 °C. Five µL of the resulting supernatant was injected into the autosampler (ShimadzuSIL-30 AC, Kyoto, Japan) for analysis. A biphenyl column (2.7 µM particle size, 2.1 mm inner diameter, 100 mm length, Restek Corporation, Bellefonte, PA, USA) was utilized to separate *p*-cresol sulfate and *p*-cresol glucuronide using an isocratic condition with a flow rate of 0.3 mL/min (at 30 °C). The mobile phase consisted of water and methanol (10:90, *v/v*) supplemented with 2 mM ammonium acetate and 0.1% formic acid. Analyte identifications were achieved with negative electrospray ionization with multiple reaction monitoring of the following mass transitions: *p*-cresol sulfate (mass to charge ratio [*m/z*]: 187.00→107.00), *p*-cresol glucuronide (*m/z*: 282.85→106.95), *p*-cresol sulfate-d_7_ (*m/z*: 194.10→114.15), and *p*-cresol glucuronide-d_7_ (*m/z*: 290.00→114.00). The assay was validated following the United States Food and Drug Administration (FDA)’s guidance document [37].

### 2.8. Quantification of p-Cresol Concentrations in HepaRG Cell Culture

An ultra-high performance liquid chromatography assay (UPLC, Shimadzu Nexera-i LC-2040 C, Kyoto, Japan) was developed and validated to quantify the concentrations of *p*-cresol in HepaRG cell culture. Thirty µL of cell supernatant or lysates was collected and deproteinated with 90 µL methanol (containing 50 µg/mL 2,6-dimethylphenol (DMP), the internal standard). The mixture was protected from light during sample extraction at room temperature (i.e., 23.5 °C) for 20 min. After extraction, the mixture was vortexed and sonicated at room temperature for 30 s and centrifuged at 4000× *g* at 4 °C for 10 min. Subsequently, the supernatant (50 µL) was injected into the autosampler and separated using the Zorbax Eclipse XDB-C18 analytical column (5 μm particle size, 4.6 mm inner diameter, 250 mm length; Agilent Technologies, Mississauga, ON, Canada) at a temperature of 40 °C. The mobile phase consisted of water and methanol (26:74, *v/v*) supplemented with 0.5 mM ammonium acetate and 0.025% formic acid at an isocratic flow rate of 0.3 mL/minute. The wavelength of the ultraviolet detector was 280 nm for both *p*-Cresol and DMP, which was pre-optimized based on control spectral scans (Shimadzu UV-2600i, Kyoto, Japan). The assay was validated following the United States Food and Drug Administration (FDA)’s guidance document [37].

### 2.9. Statistical Analysis

Only non-parametric testing with more stringent thresholds was utilized. The Mann–Whitney rank sum test was used to compare two groups, and the Kruskal–Wallis analysis of variance on ranks followed by Student–Newman–Keuls post hoc test were used to compare multiple groups (SigmaStat 3.5, Systat Software, San Jose, CA, USA) [26,27,38]. A *p* value < 0.05 was deemed a priori as the threshold for significance. The half maximal effective concentrations (EC_50_) were determined by sigmoidal 3-parameter fitting ($y\;=\frac{a}{1+\exp\left(\frac{X_{0}-X}{b}\right)}$) (SigmaPlot 14.0, Systat Software, San Jose, CA, USA). The EC_50_ values best approximated the linear portions of the dose-response curves. 1 mM of *p*-cresol was utilized in subsequent direct comparative experiments because our preliminary data had indicated that the other uremic toxins were likely less toxic than *p*-cresol (therefore, a concentration closer to the top of the EC_50_ range was utilized). On the other hand, 0.75 mM of *p*-cresol was used in subsequent chemical modulation experiments because the effects of modulation could lead to either more or less toxicity (therefore a concentration at the middle of the EC_50_ range was utilized). The exposure time (24 h) in these indicated experiments represented the in vitro condition that yielded potentially translatable physiological conditions (see Section 4).

## 3. Results

### 3.1. Positive Control and Concentration/Time-Course Responses of p-Cresol in HepaRG Cells

**Figure 2:**
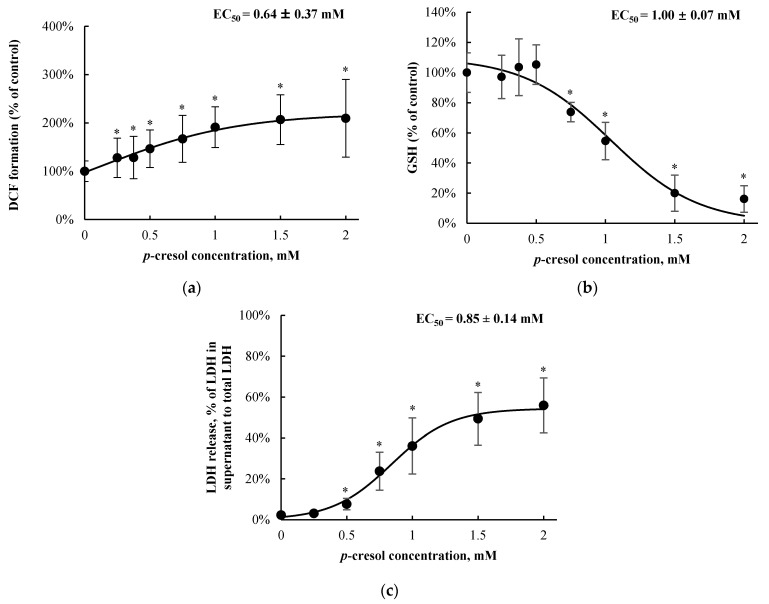
Concentration-responses of *p*-cresol on (**a**) DCF formation, (**b**) total cellular GSH depletion, and (**c**) LDH release in HepaRG cells (0.4 million cells/well). Cells were treated with 0–2 mM of *p*-cresol for 24 h as described in Section. DCF formation (*n* = 18) and total cellular GSH depletion (*n =* 12) were expressed as percentages of the vehicle control (i.e., the HepaRG differentiation medium). LDH release (*n =* 8) was calculated as the percentage of activity in the cell supernatant to that of the sum of cell supernatant and cell lysates. The EC_50_ values were calculated based on sigmoidal, 3-parameter fitting (SigmaPlot 14.0). Data are presented as mean ± standard deviation. * *p* < 0.05 versus the vehicle control using the Mann–Whitney rank sum test. DCF, 2′, 7′ –dichlorofluorescein; EC_50_, half maximal effective concentration; GSH, total cellular glutathione; LDH, lactate dehydrogenase.

**Figure 3:**
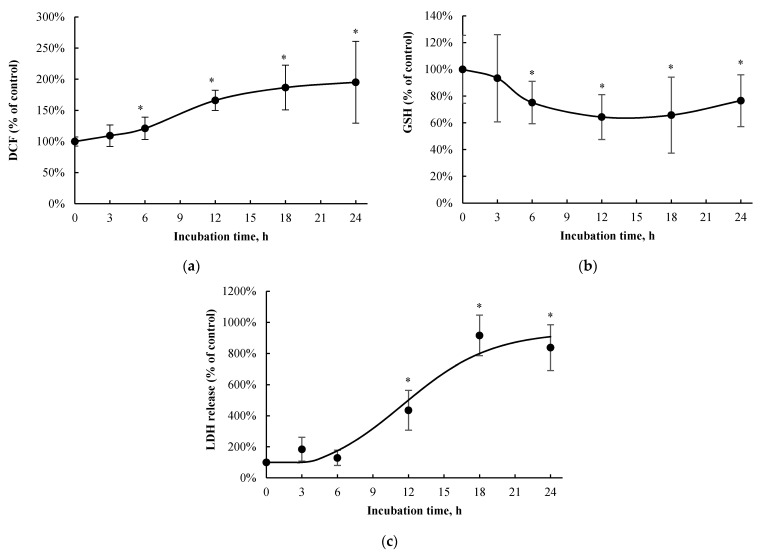
Time-dependent effects of *p*-cresol on (**a**) DCF formation, (**b**) total cellular GSH depletion, and (**c**) LDH release in HepaRG cells (0.4 million cells/well). Cells were treated with 1 mM *p*-cresol for 0, 3, 6, 12, 18, and 24 h as described in Materials and Methods. DCF formation (*n =* 12) and total cellular GSH depletion (*n =* 10) were expressed as percentages of their vehicle controls (i.e., the HepaRG differentiation medium) at corresponding treatment times. LDH release (*n =* 4) was initially calculated as the percentage of activity in the cell supernatant to that of the sum of cell supernatant and cell lysates, and further expressed as a percentage of the vehicle control at the corresponding treatment times. Data are presented as mean ± standard deviation. * *p* < 0.05 versus the vehicle control using the Mann–Whitney rank sum test. DCF, 2′, 7′ –dichlorofluorescein; GSH, total cellular glutathione; LDH, lactate dehydrogenase.

t-BOOH was utilized as an assay-based positive control for each toxicity marker [25,26,27]. t-BOOH increased DCF formation (with 5 mM, 10 min of exposure; by 297.0 ± 31.8%, *n =* 3, *p* < 0.05), decreased total cellular GSH (with 5 mM, 24 h of exposure; by 100.0 ± 0.4%, *n =* 3, *p* < 0.05), and increased LDH release (with 50 mM, 24 h of exposure; by 87.8 ± 2.7%, *n =* 3, *p* < 0.05) compared to the vehicle control, confirming the known toxic effects of the hydroperoxide in other liver cellular models (e.g., [25,26,27]). *p*-Cresol exposure resulted in concentration-dependent increases in DCF formation (EC_50_ = 0.64 ± 0.37 mM, Figure 2a), decreases in total cellular GSH concentration (EC_50_ = 1.00 ± 0.07 mM, Figure 2b), and increases in LDH release (EC_50_ = 0.85 ± 0.14 mM, Figure 2c) after 24 h of exposure. The minimum concentration of *p*-cresol causing significant toxicity was 0.25 mM (DCF formation), 0.75 mM (GSH depletion), and 0.5 mM (LDH release) (Figure 2). Based on the EC_50_ values generated from concentration-response experiments, 1 mM of *p*-cresol (representing near maximum but still linear toxicity responses) was utilized to establish the time-dependent effects for each toxicity marker. *p*-Cresol (1 mM) significantly increased DCF formation at ≥ 6 h of exposure (Figure 3a), depleted total cellular GSH at ≥ 6 h of exposure (Figure 3b), and increased LDH release at ≥ 12 h of exposure (Figure 3c), indicating possible temporal relationships between *p*-cresol-associated oxidative stress, GSH depletion, and cellular necrosis.

### 3.2. Relative Toxic Effects of p-Cresol in Comparison to Other Uremic Toxins in HepaRG Cells

**Figure 4:**
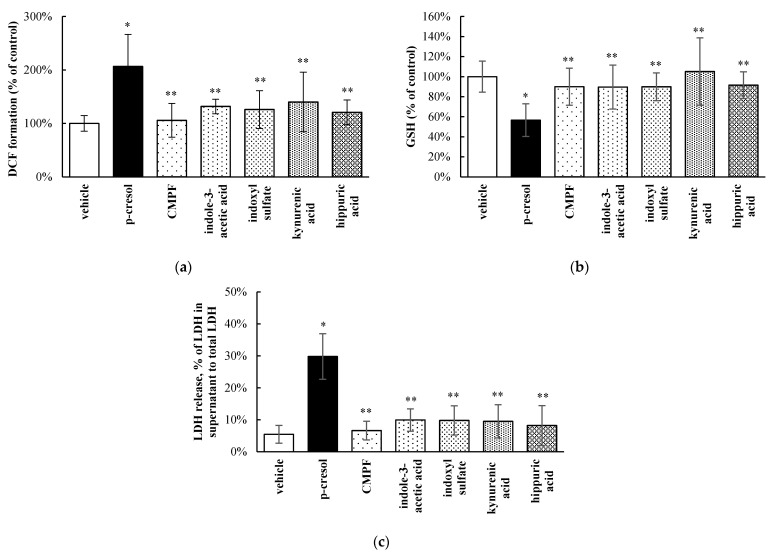
Relative effects of uremic toxins on (**a**) DCF formation, (**b**) total cellular GSH depletion, and (**c**) LDH release in HepaRG cells (0.4 million cells/well). Cells were treated with equal-molar (i.e., 1 mM) concentrations of *p*-cresol, CMPF, indole-3-acetic acid, indoxyl sulfate, kynurenic acid, or hippuric acid for 24 h as described in Section 2. DCF formation and total cellular GSH depletion were expressed as percentages of the vehicle control (i.e., the HepaRG differentiation medium). LDH release was calculated as the percentage of activity in the cell supernatant to that of the sum of cell supernatant and cell lysates. Data are presented as mean ± standard deviation from *n =* 8 determinations. * *p* < 0.05 versus the vehicle control using ANOVA on ranks; ** *p* < 0.05 versus *p*-cresol. CMPF, 3-carboxy-4-methyl-5-propyl-2-furanpropanoic acid; DCF, 2′, 7′ –dichlorofluorescein; GSH, total cellular glutathione; LDH, lactate dehydrogenase.

*p*-Cresol is a part of a large milieu of uremic toxins, and the toxic effects of *p*-cresol compared to other toxicologically important protein-bound uremic solutes [2,28] have not been systematically characterized in a hepatic model. The relative effects of *p*-cresol and other protein-bound uremic toxins on the DCF, GSH, and LDH markers were determined using equal molar conditions (i.e., 1 mM exposure for 24 h). Our data indicated *p*-cresol to be the most toxic with respect to each marker compared to all tested uremic toxins in HepaRG cells (Figure 4). Overall, CMPF, indole-3-acetic acid, indoxyl sulfate, kynurenic acid, and hippuric acid had little effects on DCF increase, GSH depletion, or LDH release when compared to the vehicle control (Figure 4). While it is ideal to consider the concurrent effects of the entire uremic milieu when assessing toxicity [39], these data supported further targeted investigations of *p*-cresol as a potent uremic toxicant in this experimental model.

### 3.3. p-Cresol Sulfate and Glucuronide Concentrations in HepaRG Cells Treated with p-Cresol

**Figure 5:**
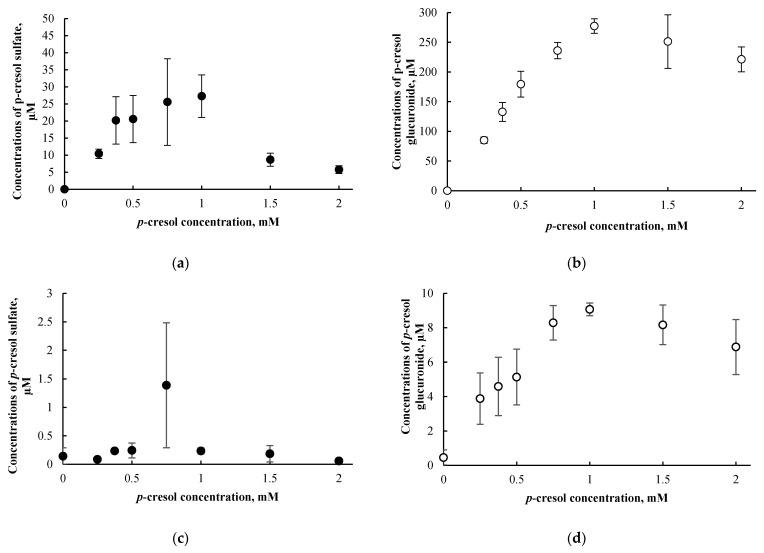
Concentration-dependent effects of *p*-cresol (0 to 2 mM, 24 h of exposure) on the formation of (**a**) *p*-cresol sulfate in culture supernatant, (**b**) *p*-cresol glucuronide in culture supernatant, (**c**) *p*-cresol sulfate in cell lysates, and (**d**) *p*-cresol glucuronide in cell lysates in HepaRG cells (0.4 million cells/well). Concentrations of *p*-cresol sulfate and *p*-cresol glucuronide were determined as described in Section 2. Data are presented as mean ± standard deviation from *n =* 3 determinations.

The formation of *p*-cresol sulfate and *p*-cresol glucuronide were approximately linear (as evident in the culture supernatant) at *p*-cresol concentrations ≤ 1000 µM after 24 h of exposure (Figure 5a,b). The maximum *p*-cresol sulfate and *p*-cresol glucuronide concentrations generated were 27.2 ± 6.2 µM and 277.3 ± 12.3 µM in the culture supernatant, respectively, achieved at 1000 µM of *p*-cresol exposure. In contrast to the culture supernatant, low concentrations of *p*-cresol sulfate and *p*-cresol glucuronide were found in the cell lysates (i.e., 1.7 ± 1.5% for *p*-cresol sulfate and 3.3 ± 1.0% for *p*-cresol glucuronide as percentages of the sum of cell lysates and supernatant from averages of all tested concentrations) (Figure 5c,d). Based on these findings, only the culture supernatant was utilized for metabolite quantification for subsequent chemical modulation experiments (please see Section 3.6 below). The observation of metabolites being found primarily in culture supernatant is consistent with other analytes in the HepaRG model [23] or in different in vitro hepatocyte models [38]. Furthermore, metabolite concentrations declined at *p*-cresol concentration > 1 mM (Figure 5), possibly due to cellular toxicity as evident by LDH release (EC_50_~1 mM, Figure 2c).

**Figure 6:**
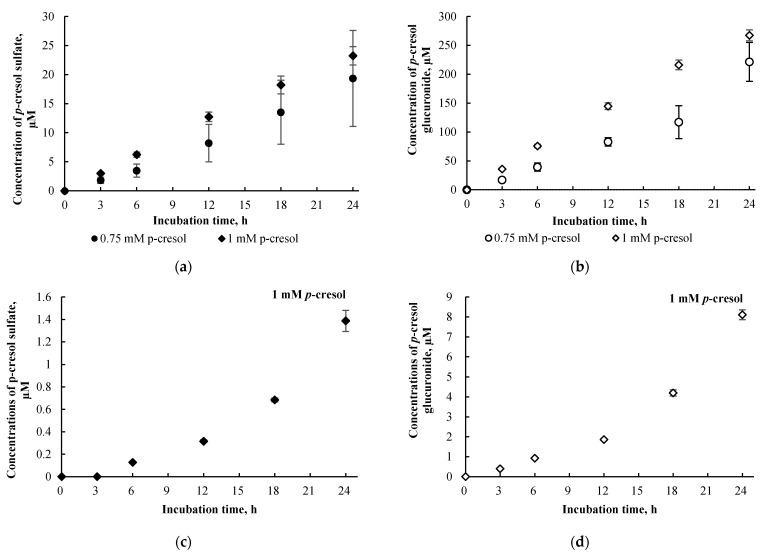
Time-dependent formations of (**a**) *p*-cresol sulfate in culture supernatant, (**b**) *p*-cresol glucuronide in culture supernatant, (**c**) *p*-cresol sulfate in cell lysates, and (**d**) *p*-cresol glucuronide in cell lysates in HepaRG cells (0.4 million cells/well) treated with 0.75 or 1 mM of *p*-cresol. Concentrations of *p*-cresol sulfate and *p*-cresol glucuronide were determined as described in Section 2. Two concentrations tested in this experiment were used for subsequent toxicity comparison and modulation experiments. Data are presented as mean ± standard deviation from *n =* 4 determinations.

Concentrations of *p*-cresol sulfate and *p*-cresol glucuronide in the culture supernatants of cells treated with 0.75 mM or 1.0 mM of *p*-cresol increased linearly as a function of incubation time from 0 to 24 h (Figure 6a,b). Consistent with our concentration-response experiments, metabolite concentrations in cell lysates were consistently low (i.e., 5.3 ± 3.7% for *p*-cresol sulfate and 3.3 ± 1.4% for *p*-cresol glucuronide as percentages of total metabolites generated in cells treated with 1.0 mM of *p*-cresol using all tested time points, Figure 6c,d); however, the increases in cell lysate metabolite concentrations were slightly curve linear. Taken together, these data confirmed the suitability of our experimental conditions (i.e., 750 µM or 1000 µM of *p*-cresol exposure for 24 h) for modulation or comparative experiments. Furthermore, *p*-cresol glucuronide was the predominant metabolite at these toxic conditions in this model (Figure 5 and Figure 6). The glucuronidation pathway was the focus of our subsequent mechanistic modulation experiments.

### 3.4. Relative Toxic Effects of p-Cresol and p-Cresol Conjugated Metabolites

**Figure 7:**
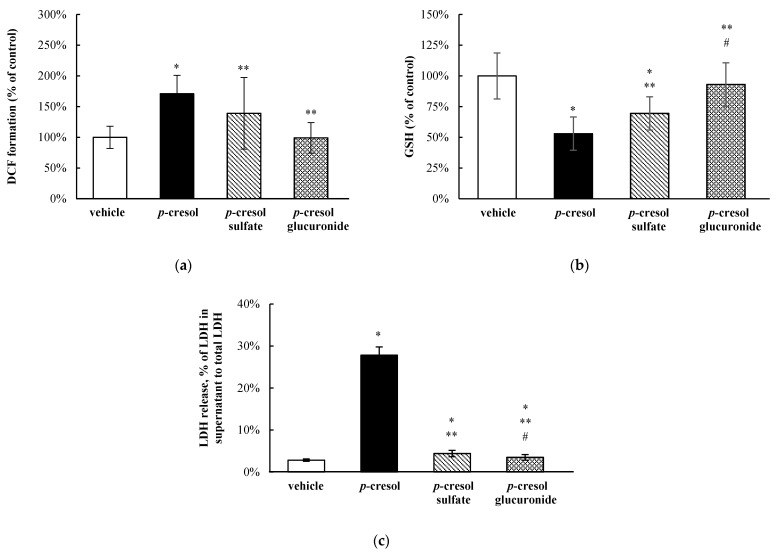
Relative effects of *p*-cresol conjugated metabolites on (**a**) DCF formation, (**b**) total cellular GSH depletion, and (**c**) LDH release in HepaRG cells (0.4 million cells/well). Cells were treated with equal-molar (i.e., 1 mM) concentrations of *p*-cresol, *p*-cresol sulfate, or *p*-cresol glucuronide for 24 h as described in Section 2. DCF formation and total cellular GSH depletion were expressed as percentages of the vehicle control (i.e., the HepaRG differentiation medium). LDH release was calculated as the percentage of activity in the cell supernatant to that of the sum of cell supernatant and cell lysates. Data are presented as mean ± standard deviation from *n =* 6 determinations. * *p* < 0.05 versus the vehicle control using ANOVA on ranks; ** *p* < 0.05 versus *p*-cresol; # *p* < 0.05 versus *p*-cresol sulfate. ANOVA, analysis of variance; DCF, 2′, 7′ –dichlorofluorescein; GSH, total cellular glutathione; LDH, lactate dehydrogenase.

At equal-molar concentrations (1 mM, 24 h of exposure), exogenously administered *p*-cresol sulfate and *p*-cresol glucuronide were less effective (i.e., less toxic) compared to *p*-cresol in generating DCF formation (by 31.9 ± 75.8% and 71.8 ± 23.8%, respectively, *p* < 0.05, Figure 7a), depleting total cellular GSH (by 16.5 ± 22.1% and 40.0 ± 19.8%, *p* < 0.05, Figure 7b), and increasing LDH release (by 23.4 ± 2.8% and 24.3 ± 1.8%, *p* < 0.05, Figure 7c). *p*-Cresol sulfate reduced total cellular GSH concentration (by 30.5 ± 13.6%, *p* < 0.05, Figure 7b), whereas both *p*-cresol sulfate and *p*-cresol glucuronide slightly increased LDH release compared to the vehicle control (Figure 7c). These findings indicated that *p*-cresol was relatively more potent than its conjugated metabolites when added exogenously at equal-molar concentrations with respect to the induction of oxidative stress, depletion of GSH, and generation of necrosis in HepaRG cells. Moreover, at these conditions, *p*-cresol sulfate was also consistently more toxic than *p*-cresol glucuronide (Figure 7).

### 3.5. p-Cresol Sulfate and Glucuronide Concentrations in HepaRG cells Treated Exogenously with p-Cresol Sulfate and p-Cresol Glucuronide

**Figure 8:**
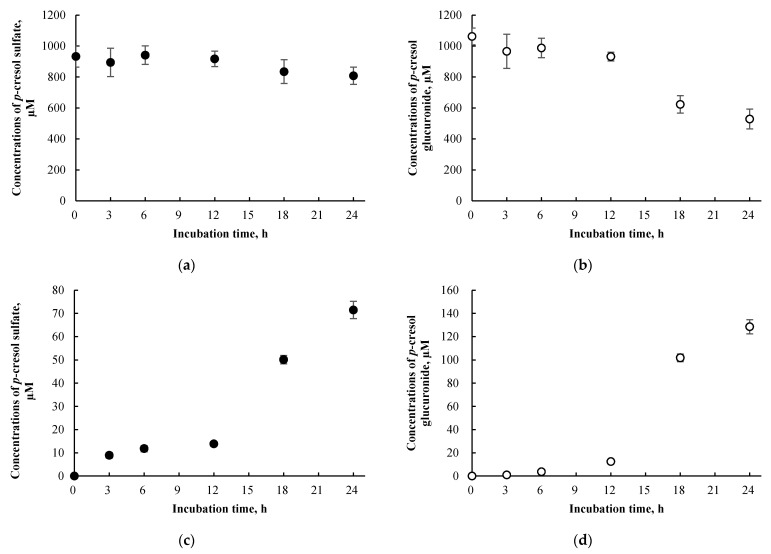
Concentrations of (**a**) *p*-cresol sulfate in culture supernatant, (**b**) *p*-cresol glucuronide in culture supernatant, (**c**) *p*-cresol sulfate in cell lysates, and (**d**) *p*-cresol glucuronide in cell lysates of HepaRG cells (0.4 million cells/well) treated with 1 mM *p*-cresol sulfate or *p*-cresol glucuronide from 0 to 24 h. Concentrations of *p*-cresol sulfate and *p*-cresol glucuronide were determined in the culture supernatant and cell lysates as described in 2. Materials and Methods. Data are presented as mean ± standard deviation from *n =* 3 determinations.

The concentrations of *p*-cresol sulfate and *p*-cresol glucuronide in cells treated exogenously with 1 mM of *p*-cresol sulfate or *p*-cresol glucuronide were compared to the concentrations of these metabolites generated in cells treated with 1 mM of *p*-cresol, in a time-dependent experiment. Concentrations of *p*-cresol sulfate and *p*-cresol glucuronide in the culture supernatant declined over treatment time (Figure 8a,b) but progressively increased in cell lysates (Figure 8c,d). These findings indicate evidence of cellular uptake with the attainment of maximum concentrations at 24 h of exposure for both *p*-cresol sulfate (71.4 ± 3.7 µM) and *p*-cresol glucuronide (128.4 ± 6.1 µM). Compared to concentrations of *p*-cresol sulfate and *p*-cresol glucuronide generated in situ in cells treated with 1 mM *p*-cresol for 24 h (Figure 6c,d), the intracellular concentrations of these metabolites obtained from exogenously administered *p*-cresol sulfate and *p*-cresol glucuronide were much higher at ~50-fold (*p*-cresol sulfate) and ~16-fold (*p*-cresol glucuronide). However, despite generating significantly higher intracellular concentrations of these metabolites, exogenously administered *p*-cresol sulfate and (especially) *p*-cresol glucuronide were significantly less toxic on all markers compared to *p*-cresol, as shown in Figure 7.

### 3.6. Determination of Conditions for the Selective Attenuation of p-Cresol Glucuronide Formation in HepaRG Cells Using Chemical Inhibitors

**Figure 9:**
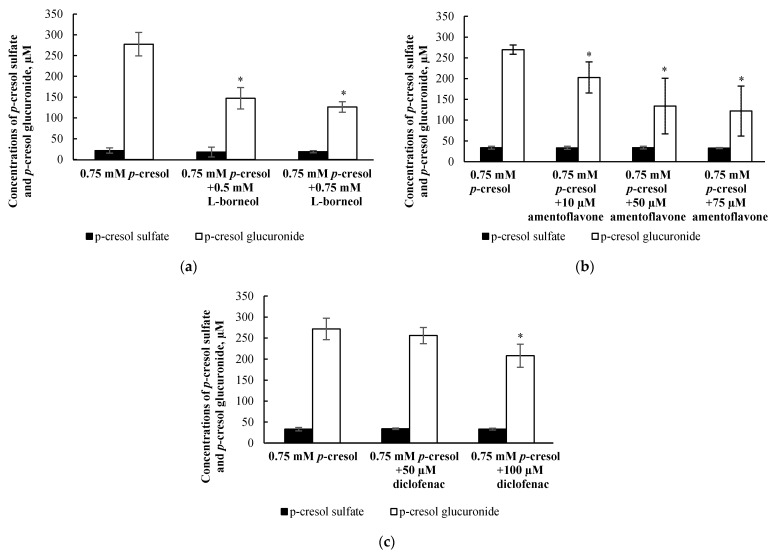
Effects of (**a**) l-borneol, (**b**) amentoflavone, and (**c**) diclofenac on the formation of *p*-cresol sulfate and *p*-cresol glucuronide in HepaRG cells (0.4 million cells/well). Cells were pre-treated with the vehicle, and non-toxic concentrations of l-borneol (0.5, 0.75 mM), amentoflavone (10, 50, 75 μM), or diclofenac (50, 100 μM) for 30 min before co-treatment with 0.75 mM *p*-cresol for 24 h. Data are presented as mean ± standard deviation from *n =* 8 determinations. The percentages of inhibition by 0.75 mM l-borneol, 75 μM amentoflavone, and 100 μM diclofenac were 53.9 ± 8.2%, 54.6 ± 22.7% and 22.5 ± 14.9% compared to the control (*p*-cresol treatment), respectively. * *p* < 0.05 versus *p*-cresol control using ANOVA on ranks. ANOVA, analysis of variance.

The formation of *p*-cresol glucuronide in the human liver is primarily catalyzed by UGT1A6, with minor contribution from UGT1A9 [32]. In order to attenuate the production of in situ-generated *p*-cresol glucuronide, multiple inhibitors with different mechanisms of actions were utilized, including l-borneol (as UDPGA depleting agent), amentoflavone (as UGT1A6 inhibitor), and diclofenac (as UGT1A6 inhibitor) [29,30,31,32]. Our preliminary experiment exposed HepaRG cells to multiple concentrations of each inhibitor alone for 24.5 h (i.e., 0.5 h pre-treatment and 24 h co-treatment) and found 1 mM l-borneol (6.0 ± 1.2%, *n =* 9), 100 µM amentoflavone (4.2 ± 1.5%, *n =* 3), and 200 µM diclofenac (5.3 ± 3.1%, *n =* 9) to slightly increase LDH release, indicating the manifestation of low grade toxicity, compared to the vehicle control (2.1 ± 0.8%, *n =* 9). Therefore, our subsequent experiments tested the effects of non-toxic concentrations of l-borneol (i.e., 0.5 and 0.75 mM), amentoflavone (i.e., 10, 50, 75 µM), and diclofenac (i.e., 50 and 100 µM) on *p*-cresol glucuronide and *p*-cresol sulfate formation, with the objective of achieving a balance between maximum attainable inhibition of *p*-cresol glucuronide formation without affecting *p*-cresol sulfate concentrations or generating cellular necrosis (LDH). Our findings indicated that l-borneol (0.75 mM), amentoflavone (75 µM) and diclofenac (100 µM) were the optimal inhibitor conditions in this model as they selectively reduced the concentration of *p*-Cresol glucuronide by 151.2 ± 37.2 µM (i.e., by 53.9 ± 8.2%), 147.7 ± 62.4 µM (by 54.6 ± 22.7%), and 63.7 ± 44.8 µM (by 22.5 ± 14.9%), compared to the control (i.e., 0.75 mM *p*-cresol), respectively, without affecting concentrations of *p*-cresol sulfate (Figure 9).

### 3.7. Effects of l-Borneol, Amentoflavone, or Diclofenac on p-Cresol Generated Cellular Necrosis

**Figure 10:**
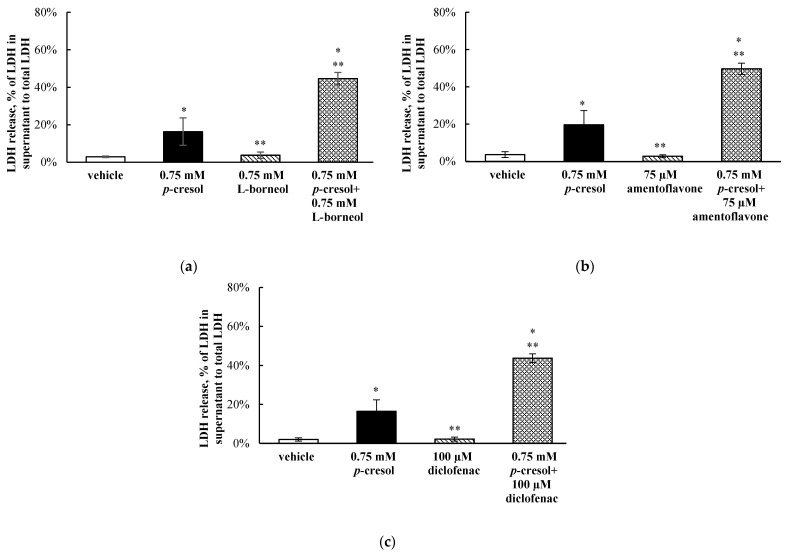
Effects of (**a**) l-borneol, (**b**) amentoflavone, and (**c**) diclofenac on *p*-cresol-mediated LDH release in HepaRG cells (0.4 million cells/well). Cells were pre-treated with vehicle or 0.75 mM l-borneol, 75 μM, amentoflavone, or 100 μM diclofenac for 30 min, then co-treated with vehicle or 0.75 mM *p*-cresol for 24 h as described in Section 2. LDH release was calculated as the percentage of activity in the cell supernatant to that of the sum of cell supernatant and cell lysates. Data are presented as mean ± standard deviation from *n =* 8 determinations. * *p* < 0.05 versus the vehicle control (i.e., the HepaRG differentiation medium) using ANOVA on ranks; ** *p* < 0.05 versus *p*-Cresol. ANOVA, analysis of variance; LDH, lactate dehydrogenase.

To determine the role of in situ-generated *p*-cresol glucuronide in mediating the toxicities of *p*-cresol, cells were exposed to the vehicle, *p*-cresol, chemical inhibitor (l-borneol, amentoflavone, or diclofenac), or *p*-cresol with each individual chemical inhibitor (at non-toxic inhibitor conditions characterized to be selectively inhibitory toward the glucuronidation of *p*-Cresol, please see Section 3.6). The LDH marker was the primary toxicity endpoint in this mechanistic experiment because it reflected the ultimate cellular toxicity outcome in this model as evident in our time-course experiments (Figure 3) and that the chemical modulators were only optimized based on this specific marker. *p*-Cresol at 0.75 mM was utilized in this experiment as it reflected the EC_50_ values of *p*-cresol-induced LDH release (Figure 2c) and generated linear metabolite formations (Figure 5 and Figure 6). l-borneol (0.75 mM), amentoflavone (75 µM) or diclofenac (100 µM) alone did not affect LDH release (vs. vehicle control), but each inhibitor independently increased *p*-cresol-mediated LDH release by 28.3 ± 5.3%, 30.0 ± 8.2% or 27.3 ± 6.8%, respectively, compared to *p*-cresol treatment (Figure 10).

### 3.8. Effects of l-Borneol-borneol, Amentoflavone, or Diclofenac on Cellular p-Cresol Concentrations

**Figure 11:**
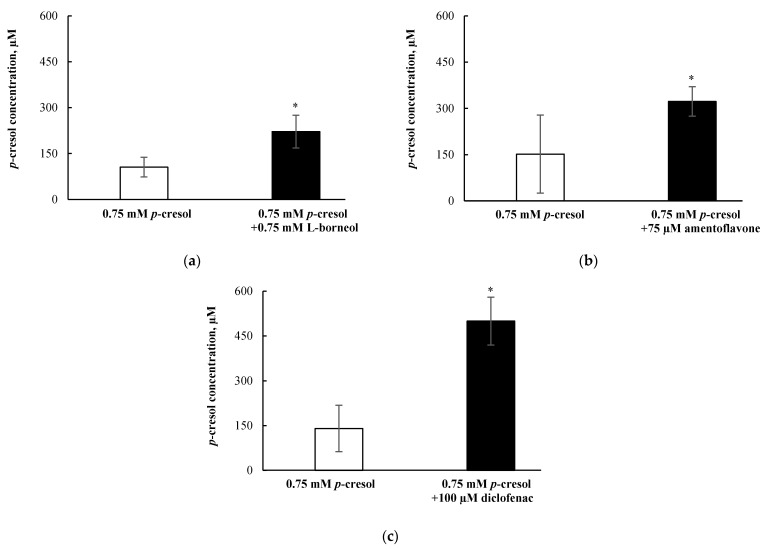
Effects of (**a**) l-borneol, (**b**) amentoflavone, and (**c**) diclofenac on *p*-cresol concentrations in the supernatant of HepaRG cells (0.4 million cells/well). Cells were pre-treated with 0.75 mM l-borneol, 75 μM amentoflavone, or 100 μM diclofenac for 30 min, then co-treated with vehicle or 0.75 mM *p*-cresol for 24 h. *p*-cresol concentrations were quantified as described in Materials and Methods section. Data are presented as mean ± standard deviation from *n =* 8 determinations. * *p* < 0.05 versus *p*-cresol control using the Mann–Whitney rank sum test.

The selective attenuation (i.e., without affecting the sulfonation) of *p*-cresol glucuronide formation resulted in increased *p*-cresol-generated cellular toxicity (Figure 10); therefore, it was pertinent to determine if the loss of glucuronidation corresponded to increased *p*-cresol concentrations under these treatment conditions. Our findings indicated that l-borneol (0.75 mM), amentoflavone (75 µM) and diclofenac (100 µM) independently increased the concentrations of *p*-cresol (characterized in the culture supernatant) by 116.1 ± 70.4 µM (i.e., by 209.9 ± 50.6%,), 171.8 ± 106.8 µM (i.e., by 212.6 ± 31.6%), and 359.3 ± 134.1 µM (i.e., by 356.0 ± 57.2%), respectively (Figure 11), indicating that the inhibition of *p*-cresol glucuronidation resulted in the accumulation of the parent compound.

### 3.9. Validation of LC/MS/MS and UPLC Assays for the Quantification of p-Cresol Sulfate, p-Cresol Glucuronide, and p-Cresol

The LC/MS/MS and UPLC assays used to quantify *p*-cresol sulfate, *p*-cresol glucuronide, and *p*-cresol were validated following the United States Food and Drug Administration (FDA)’s guidance document [37]. For the measurement of *p*-cresol sulfate and *p*-cresol glucuronide on the LC/MS/MS, the calibration curves were linear between 0.001 ng/mL and 80 µg/mL, and 0.08 and 80 µg/mL, respectively (Supplementary Materials, Figures S1 and S2). The total run time was 5 min. The bias and imprecision of high-, mid-, and low-quality control samples were <15% of the nominal concentrations, and that of the lower limit of quantitation quality control samples were <20% (Supplementary Materials, Table S1). The autosampler, bench-top, freeze-thaw, and two-week storage stabilities were all within 15% with respect to bias determination using high- and low-quality control samples (Supplementary Materials, Table S2). For the measurement of *p*-cresol on the UPLC, the calibration curve was linear between 5 and 320 µg/mL (Supplementary Materials, Figure S3). The run time was 15 min. The bias and imprecision of all quality control samples were <15% of the nominal concentrations (Supplementary Materials, Table S3). The autosampler, bench-top, freeze-thaw, and long-term stabilities with respect to bias determination using high- and low-quality control samples were all < 15% (Supplementary Materials, Table S4). Overall, all validation parameters passed the criteria suggested by the FDA [37].

## 4. Discussion

*p*-Cresol generated concentration- and time-dependent responses on markers of oxidative stress, total cellular glutathione, and cellular necrosis in HepaRG cells. Although the toxic effects of *p*-cresol have been reported in various in vitro or in vivo animal liver models [14,15,16,17,40], the observations of direct toxicological effects (Figure 2 and Figure 3) in a human hepatic model are, to our knowledge, novel findings. The effects of *p*-cresol on oxidative stress induction (i.e., DCF formation, Figure 2a and Figure 3a), glutathione depletion (Figure 2b and Figure 3b), and cellular necrosis (Figure 2c and Figure 3c) were consistent with its effects in other models: DCF generation in human umbilical vein endothelial and U937 mononuclear cells (e.g., [41,42]); glutathione depletion in rat liver slices or glutathione adduct formation in GSH-fortified human liver microsomes (e.g., [16,19]); and LDH release in rat liver slices, human colonic epithelial cells, and human bone marrow-derived mesenchymal stem cells (e.g., [16,43,44]). The EC_50_ values associated with *p*-cresol exposure observed of these markers in HepaRG cells (Figure 2) could be considered physiologically attainable under toxic conditions in humans. This is based on the documentation of relatively elevated plasma concentrations of *p*-cresol metabolites in hemodialysis patients as high as ~1.66 mM [36], which may indirectly infer that similar toxic concentrations of the precursor *p*-cresol can be attainable in the liver, the primary organ of *p*-cresol metabolism and hence the direct origin of these metabolites [4]. Temporal experiments suggested the time-dependent relationships between *p*-cresol-induced oxidative injury (initially evident at 6 h of exposure), glutathione depletion (6 h), and the eventual manifestation of cellular necrosis (12 h) (Figure 3). Although the role of cellular glutathione in mediating *p*-cresol-induced LDH release has been demonstrated in rat liver slices [16], further mechanistic experiments (not part of current objectives) are required to establish cause–effect relationships of these toxicity markers from *p*-cresol exposure in this human model. Furthermore, *p*-cresol was relatively more potent on each toxicity marker compared to a sample panel of toxicologically relevant protein-bound uremic solutes (Figure 4), supporting a targeted mechanistic investigation on *p*-cresol alone in this study. The lack of substantial increases in DCF formation by indole-3-acetic acid, indoxyl-sulfate, kynurenic acid, and hippuric in our model (Figure 4a) was consistent with that reported by Weigand et al. [13] in primary cultures of human hepatocytes. However, the usage of different markers of toxicity, under different exposure conditions, in different liver cell types, and in the absence of *p*-cresol control [13] precluded further direct comparisons between the two studies.

Both concentration-response (Figure 5) and time-course (Figure 6) experiments indicated *p*-cresol glucuronide to be the predominant, and *p*-cresol sulfate a relatively minor, in situ-generated metabolite from *p*-cresol exposure in HepaRG cells. While the relative concentrations of these two metabolites in our model were inconsistent with that observed in the human plasma (i.e., higher sulfate than glucuronide based on total concentrations) under typical uremic conditions (e.g., [5,6,7,8,9,10]), this observation might be explained by the kinetic behaviors of the associated enzymes. Recently, it was determined that UGT1A6 was the primary enzyme contributing to the production *p*-cresol glucuronide in human liver microsomes [32] and sulfotransferase (SULT)1A1 as the predominant enzyme responsible for *p*-cresol sulfation in human liver cytosols [45]. The UGT1A6-mediated *p*-cresol glucuronidation in human liver microsomes [32] has a much lower affinity (i.e., substrate concentration at half maximum reaction rate, K_m_ = 67.3 ± 17.3 µM) and higher capacity (i.e., maximum reaction rate, V_max_ = 8.5 ± 0.7 nmol/mg/min) compared to SULT1A1-mediated *p*-cresol sulfate formation in human liver cytosols [45], which is consistent with the general kinetic behaviors of UGT and SULT enzymes [46,47]. Therefore, under conditions of high *p*-cresol concentrations required to generate toxicity in our model, the glucuronidation pathway was likely preferred over sulfonation. This general kinetic behavior is also evident in clinical studies where Poesen et al. [5] and Mutsaers et al. [7] both independently illustrated a shift from *p*-cresol sulfate to *p*-cresol glucuronide production in patients with more severe stages of kidney disease, possibly due to the accumulation of *p*-cresol. Furthermore, the level of constitutive gene expressions associated with UGT1A6 and SULT1A1 in HepaRG cells were comparable to human hepatocytes based on microarray analysis [48]; thus, it was unlikely that discrepancies in metabolite generation were due to significantly altered enzyme levels in our model. However, comparisons on UGT1A6/SULT1A1 protein expression and probe specific activities in HepaRG cells are still lacking in the literature. Collectively, these data suggested that glucuronidation is likely a quantitatively important pathway, whether it be responsible for detoxification or toxification (discussed below), in the human liver at elevated toxic concentrations of *p*-cresol.

Overall, our data indicated that *p*-cresol glucuronide or the glucuronidation pathway was likely associated with the detoxification of *p*-cresol in HepaRG cells, based on the following complementary findings: (1) Exogenously administered *p*-cresol glucuronide was significantly less toxic than *p*-cresol at equal molar conditions (Figure 7), despite generating much higher concentrations of intracellular *p*-cresol glucuronide (Figure 8d) than *p*-cresol (Figure 6d). (2) Selective attenuation of in situ-generated *p*-cresol glucuronide (Figure 9) resulted in significantly increased LDH release from *p*-cresol exposure (Figure 10), and these findings were reproducible using multiple chemical inhibitors with independent mechanisms of actions (i.e., l-borneol being a UDPGA co-factor depletory agent; amentoflavone and diclofenac being reversible enzyme inhibitors [29,30,31,32]). (3) The enhanced LDH release from reduced in situ *p*-cresol glucuronide formation (Figure 10) was associated with the accumulation of the parent compound, *p*-cresol (Figure 11). These observations are consistent with the general assertion that the glucuronidation reaction and glucuronide metabolites are typically associated with xenobiotic detoxification (with the exception of acyl-glucuronides) [49,50]. More specifically, these findings are consistent with observations of lack of *p*-cresol glucuronide toxicity in various human cell types: *p*-cresol glucuronide (sodium or calcium salt) up to 100 µM was generally less toxic than *p*-cresol as measured by crystal violet staining in HEK293 cells [12]; *p*-cresol glucuronide appeared less effective in reducing mitochondrial metabolism (measured by 3-4,5-dimethylthiazol-2-yl]-2,5-diphenyl tetrazolium bromide) compared to *p*-cresol in ciPTEC cells exposed to 2mM of each compound for 48 h [11]; isolated whole blood from healthy human volunteers exposed to 48 mg/L of *p*-cresol glucuronide alone for 10 min did not exhibit enhanced oxidative burst activities compared to the vehicle control [10]; and *p*-cresol glucuronide (500 µM) had minimal or no effects on mitochondrial membrane potential, lactate production, or reactive oxygen species production in primary cultures of human hepatocytes with 96 h of exposure [13]. Although *p*-cresol glucuronide was effective in reducing hepatocyte viability and cellular ATP [13], comparative effects to *p*-cresol were not established in their model.

The lack of toxicity associated with *p*-cresol glucuronide formation in our model could suggest alternative pathway(s) of *p*-cresol metabolism (or metabolites) may be involved in mediating the toxicity of *p*-cresol. *p*-Cresol is known to undergo cytochrome P450 (CYP)-mediated oxidative metabolism in the production of reactive intermediates as demonstrated in GSH-fortified human liver microsomes [19] and rat liver slices [16,17]. The potential role of toxic oxidative metabolites was demonstrated in rat liver slices where phenobarbital animal pre-treatment further increased cellular toxicity (measured by loss of potassium) associated with *p*-cresol treatment, and the effects were reduced by metyrapone, a CYP450 inhibitor [16]. The consequence of reactive intermediates was suggested by the depletion of cellular glutathione in *p*-cresol-exposed rat liver slices [16], which was consistent with our observation of *p*-cresol associated concentration- and time-dependent reductions in total cellular glutathione in HepaRG cells (Figure 2b and Figure 3b). However, the metabolite and *p*-cresol concentration data obtained in our model could potentially argue against the hypothesis of toxic CY*P*-generated metabolites (Figure 9 and Figure 11). Specifically, the decrease in *p*-cresol glucuronide by l-borneol (a relatively specific glucuronidation inhibitor) corresponded with roughly equal increases in *p*-cresol concentration (Figure 9a and Figure 11a), but higher increases in *p*-cresol concentrations compared to reductions in glucuronide concentrations were observed for both amentoflavone and diclofenac (Figure 9b,c and Figure 11b,c), which may be explained by the additional inhibitory effects of these two chemicals toward CYP enzymes which may mediate the metabolism of *p*-cresol [19,30,51,52]. On the other hand, although amentoflavone and diclofenac inhibited multiple UGT enzymes [30,31], their non-specific effects toward glucuronidation were unlikely to have contributed to this observation since *p*-cresol was primarily conjugated by a single UGT enzyme in the human liver (i.e., UGT1A6) [32]. However, despite potential evidence of CYP450 inhibition by amentoflavone and diclofenac in our model, all three chemical modulators affected *p*-cresol-mediated LDH release to the same extent (Figure 10), suggesting that the possible CYP inhibition by amentoflavone and diclofenac did not reduce *p*-cresol-generated toxicity in our model. Therefore, further control experiments (i.e., measurement of probe substrate activities and use of selective CYP450 modulators) are needed to test the toxic oxidative metabolism hypothesis. Furthermore, despite data demonstrating exogenously administered *p*-cresol sulfate was less toxic than *p*-cresol in HepaRG cells (Figure 7), *p*-cresol sulfate was consistently more toxic than the glucuronide (Figure 7). Therefore, further mechanistic experiments testing the in situ production of this metabolite in relation to the manifestation of toxicity are also warranted, given the significant body of literature supporting the toxicology of *p*-cresol sulfate in various clinical and experimental models [2,3,4].

Our findings should be considered in the context of the following limitations: (1) It was necessary for direct comparative purposes to utilize relatively high concentrations of metabolites or other uremic solutes in parts of our study (i.e., consistent with the approach of other investigators in this area (e.g., [7,12,13]), which may have exceeded typical physiological concentrations; however, these were supported by further mechanistic studies involving metabolites generated in situ from *p*-cresol exposure (Figure 9, Figure 10 and Figure 11). (2) While many of the recommendations proposed by the EUTox group for conducting in vitro studies involving uremic toxins [39] have been considered for this investigation, some suggested conditions may not be relevant to this model. For example, our model [21,22,24] had already been optimized for the generation of albumin and therefore it was not necessary, and may have been harmful to the cells, to add 35 g/L of albumin to the incubation medium. (3) The chemical inhibition approach in our mechanistic study was not specific (e.g., both amentoflavone and diclofenac having potential effects on CYP450 oxidation). However, the usage of three independent inhibitors provided consistent findings with respect to the primary toxicity endpoint (e.g., LDH release), suggesting the robustness of our data. (4) The apparent lack of toxicity of exogenously administered or in situ-generated *p*-cresol glucuronide may be a specific observation in this hepatic model and should be further verified in other target tissues of *p*-cresol toxicity (i.e., heart and kidney cells).

## 5. Conclusions

In conclusion, our novel findings indicate that *p*-cresol can induce oxidative stress, glutathione depletion, and cellular necrosis at toxic concentrations potentially obtainable in humans; that *p*-cresol was relatively more potent than other widely studied uremic toxins; and the formation of *p*-cresol glucuronide was unlikely to be associated with the manifestation of its toxic effects in HepaRG cells. Further experiments are being conducted to test the roles of CYP450 oxidation and sulfonation in *p*-cresol mediated toxicity in this model.

## Data Availability

The data presented in this study are available on request from the corresponding author (Tony Kiang).

## Supplementary Materials

The following are available online at https://www.mdpi.com/article/10.3390/pharmaceutics13060857/s1, Figure S1: Calibration curve of *p*-cresol sulfate based on a weighted (1/x^2^) least-squares regression model, Figure S2: Calibration curve of *p*-cresol glucuronide based on a weighted (1/x^2^) least-squares regression model, Figure S3: Calibration curve of *p*-cresol based on a weighted (1/x^2^) least-squares regression model, Table S1: Accuracy and precision data of the UPLC/MS/MS assay for the measurement of *p*-cresol sulfate and *p*-cresol glucuronide, Table S2: Stability data of the UPLC/MS/MS assay for the measurement of *p*-cresol sulfate and *p*-cresol glucuronide, Table S3: Accuracy and precision data of the UPLC assay for the measurement of *p*-cresol, Table S4: Stability data of the UPLC assay for the measurement of *p*-cresol.

## References

[1] Vanholder R., De Smet R., Lesaffer G. (1999). p-Cresol: A Toxin Revealing Many Neglected but Relevant Aspects of Uraemic Toxicity. Nephrol. Dial. Transplant..

[2] Vanholder R., Pletinck A., Schepers E., Glorieux G. (2018). Biochemical and Clinical Impact of Organic Uremic Retention Solutes: A Comprehensive Update. Toxins.

[3] Glorieux G., Vanholder R., Van Biesen W., Pletinck A., Schepers E., Neirynck N., Speeckaert M., De Bacquer D., Verbeke F. (2021). Free p-Cresyl Sulfate shows the Highest Association with Cardiovascular Outcome in Chronic Kidney Disease. Nephrol. Dial. Transplant..

[4] Gryp T., Vanholder R., Vaneechoutte M., Glorieux G. (2017). p-Cresyl Sulfate. Toxins.

[5] Poesen R., Evenepoel P., de Loor H., Kuypers D., Augustijns P., Meijers B. (2016). Metabolism, Protein Binding, and Renal Clearance of Microbiota-Derived p-Cresol in Patients with CKD. Clin. J. Am. Soc. Nephrol..

[6] Liabeuf S., Glorieux G., Lenglet A., Diouf M., Schepers E., Desjardins L., Choukroun G., Vanholder R., Massy Z.A. (2013). European Uremic Toxin (EUTox) Work Group. Does p-Cresyl Glucuronide have the Same Impact on Mortality as Other Protein-Bound Uremic Toxins?. PLoS ONE.

[7] Mutsaers H.A., Caetano-Pinto P., Seegers A.E., Dankers A.C., van den Broek P.H., Wetzels J.F., van den Brand J.A., van den Heuvel L.P., Hoenderop J.G., Wilmer M.J. (2015). Proximal Tubular Efflux Transporters Involved in Renal Excretion of p-cresyl Sulfate and p-Cresyl Glucuronide: Implications for Chronic Kidney Disease Pathophysiology. Toxicol. In Vitro.

[8] Itoh Y., Ezawa A., Kikuchi K., Tsuruta Y., Niwa T. (2012). Protein-Bound Uremic Toxins in Hemodialysis Patients Measured by Liquid Chromatography/Tandem Mass Spectrometry and their Effects on Endothelial ROS Production. Anal. Bioanal Chem..

[9] Chinnappa S., Tu Y.K., Yeh Y.C., Glorieux G., Vanholder R., Mooney A. (2018). Association between Protein-Bound Uremic Toxins and Asymptomatic Cardiac Dysfunction in Patients with Chronic Kidney Disease. Toxins.

[10] Meert N., Schepers E., Glorieux G., Van Landschoot M., Goeman J.L., Waterloos M.A., Dhondt A., Van der Eycken J., Vanholder R. (2012). Novel Method for Simultaneous Determination of p-Cresyl Sulphate and p-Cresyl Glucuronide: Clinical Data and Pathophysiological Implications. Nephrol. Dial. Transplant..

[11] Mutsaers H.A., Wilmer M.J., Reijnders D., Jansen J., van den Broek P.H., Forkink M., Schepers E., Glorieux G., Vanholder R., van den Heuvel L.P. (2013). Uremic Toxins Inhibit Renal Metabolic Capacity through Interference with Glucuronidation and Mitochondrial Respiration. Biochim. Biophys. Acta.

[12] London J.A., Wang E.C.S., Barsukov I.L., Yates E.A., Stachulski A.V. (2020). Synthesis and Toxicity Profile in 293 Human Embryonic Kidney Cells of the Β D-Glucuronide Derivatives of Ortho-, Meta- and Para-Cresol. Carbohydr. Res..

[13] Weigand K.M., Schirris T.J.J., Houweling M., van den Heuvel J.J.M.W., Koenderink J.B., Dankers A.C.A., Russel F.G.M., Greupink R. (2019). Uremic Solutes Modulate Hepatic Bile Acid Handling and Induce Mitochondrial Toxicity. Toxicol. In Vitro.

[14] Kitagawa A. (2001). Effects of Cresols (O-, M-, and P-Isomers) on the Bioenergetic System in Isolated Rat Liver Mitochondria. Drug Chem. Toxicol..

[15] Abreo K., Sella M., Gautreaux S., De Smet R., Vogeleere P., Ringoir S., Vanholder R. (1997). P-Cresol, a Uremic Compound, Enhances the Uptake of Aluminum in Hepatocytes. J. Am. Soc. Nephrol..

[16] Thompson D.C., Perera K., Fisher R., Brendel K. (1994). Cresol Isomers: Comparison of Toxic Potency in Rat Liver Slices. Toxicol. Appl. Pharmacol..

[17] Thompson D.C., Perera K., London R. (1996). Studies on the Mechanism of Hepatotoxicity of 4-Methylphenol (p-Cresol): Effects of Deuterium Labeling and Ring Substitution. Chem. Biol. Interact..

[18] Ikematsu N., Kashiwagi M., Hara K., Waters B., Matsusue A., Takayama M., Kubo S.I. (2019). Organ Distribution of Endogenous p-Cresol in Hemodialysis Patients. J. Med. Investig..

[19] Yan Z., Zhong H.M., Maher N., Torres R., Leo G.C., Caldwell G.W., Huebert N. (2005). Bioactivation of 4-Methylphenol (p-Cresol) Via Cytochrome P450-Mediated Aromatic Oxidation in Human Liver Microsomes. Drug Metab. Dispos..

[20] Ashraf M.N., Asghar M.W., Rong Y., Doschak M.R., Kiang T.K.L. (2019). Advanced in Vitro HepaRG Culture Systems for Xenobiotic Metabolism and Toxicity Characterization. Eur. J. Drug Metab. Pharmacokinet..

[21] Gripon P., Rumin S., Urban S., Le Seyec J., Glaise D., Cannie I., Guyomard C., Lucas J., Trepo C., Guguen-Guillouzo C. (2002). Infection of a Human Hepatoma Cell Line by Hepatitis B Virus. Proc. Natl. Acad. Sci. USA.

[22] Guillouzo A., Corlu A., Aninat C., Glaise D., Morel F., Guguen-Guillouzo C. (2007). The Human Hepatoma HepaRG Cells: A Highly Differentiated Model for Studies of Liver Metabolism and Toxicity of Xenobiotics. Chem. Biol. Interact..

[23] Rong Y., Kiang T.K.L. (2019). Development and Validation of a Sensitive Liquid-Chromatography Tandem Mass Spectrometry Assay for Mycophenolic Acid and Metabolites in HepaRG Cell Culture: Characterization of Metabolism Interactions between p-Cresol and Mycophenolic Acid. Biomed. Chromatogr..

[24] Biopredic International (2016). HepaRG Cell Line Specifications. https://www.biopredic.com/.

[25] Liang F., Fang Y., Cao W., Zhang Z., Pan S., Xu X. (2018). Attenuation of Tert-Butyl Hydroperoxide (T-BHP)-Induced Oxidative Damage in HepG2 Cells by Tangeretin: Relevance of the Nrf2-ARE and MAPK Signaling Pathways. J. Agric. Food Chem..

[26] Kiang T.K., Teng X.W., Karagiozov S., Surendradoss J., Chang T.K., Abbott F.S. (2010). Role of Oxidative Metabolism in the Effect of Valproic Acid on Markers of Cell Viability, Necrosis, and Oxidative Stress in Sandwich-Cultured Rat Hepatocytes. Toxicol. Sci..

[27] Kiang T.K., Teng X.W., Surendradoss J., Karagiozov S., Abbott F.S., Chang T.K. (2011). Glutathione Depletion by Valproic Acid in Sandwich-Cultured Rat Hepatocytes: Role of Biotransformation and Temporal Relationship with Onset of Toxicity. Toxicol. Appl. Pharmacol..

[28] Prokopienko A.J., Nolin T.D. (2018). Microbiota-Derived Uremic Retention Solutes: Perpetrators of Altered Nonrenal Drug Clearance in Kidney Disease. Expert Rev. Clin. Pharmacol..

[29] Watkins J.B., Klaassen C.D. (1983). Chemically-Induced Alteration of UDP-Glucuronic Acid Concentration in Rat Liver. Drug Metab. Dispos..

[30] Lv X., Zhang J.B., Wang X.X., Hu W.Z., Shi Y.S., Liu S.W., Hao D.C., Zhang W.D., Ge G.B., Hou J. (2018). Amentoflavone is a Potent Broad-Spectrum Inhibitor of Human UDP-Glucuronosyltransferases. Chem. Biol. Interact..

[31] Uchaipichat V., Mackenzie P.I., Guo X.H., Gardner-Stephen D., Galetin A., Houston J.B., Miners J.O. (2004). Human Udp-Glucuronosyltransferases: Isoform Selectivity and Kinetics of 4-Methylumbelliferone and 1-Naphthol Glucuronidation, Effects of Organic Solvents, and Inhibition by Diclofenac and Probenecid. Drug Metab. Dispos..

[32] Rong Y., Kiang T.K.L. (2020). Characterizations of Human UDP-Glucuronosyltransferase Enzymes in the Conjugation of p-Cresol. Toxicol. Sci..

[33] Chen X., Zhong Z., Xu Z., Chen L., Wang Y. (2010). 2′,7′-Dichlorodihydrofluorescein as a Fluorescent Probe for Reactive Oxygen Species Measurement: Forty Years of Application and Controversy. Free Radic. Res..

[34] Cayman Chemical (2016). Glutathione Assay Kit Booklet. https://www.caymanchem.com/product/703002/glutathione-assay-kit.

[35] Roche (2016). Cytotoxicity Detection Kit (LDH) Protocol. https://www.sigmaaldrich.com/content/dam/sigma-aldrich/docs/roche/bulletin/1/11644793001bul.pdf.

[36] Cuoghi A., Caiazzo M., Bellei E., Monari E., Bergamini S., Palladino G., Ozben T., Tomasi A. (2012). Quantification of p-Cresol Sulphate in Human Plasma by Selected Reaction Monitoring. Anal. Bioanal Chem..

[37] US Food and Drug Administration (2018). Bioanalytical Method Validation Guidance for Industry. https://www.fda.gov/files/drugs/published/bioanalytical-method-validation-guidance-for-industry.pdf.

[38] Surendradoss J., Chang T.K., Abbott F.S. (2014). Evaluation of in Situ Generated Valproyl 1-O-Beta-Acyl Glucuronide in Valproic Acid Toxicity in Sandwich-Cultured Rat Hepatocytes. Drug Metab. Dispos..

[39] Cohen G., Glorieux G., Thornalley P., Schepers E., Meert N., Jankowski J., Jankowski V., Argiles A., Anderstam B., Brunet P. (2007). Review on Uraemic Toxins III: Recommendations for Handling Uraemic Retention Solutes in Vitro—Towards a Standardized Approach for Research on Uraemia. Nephrol. Dial. Transplant..

[40] U.S. Environmental Protection Agency (2010). Provisional Peer-Reviewed Toxicity Values for 4-Methylphenol (p-Cresol). https://Cfpub.epa.gov/ncea/pprtv/documents/Methylphenol4.pdf.

[41] Sheu J.J., Yang C.C., Wallace C.G., Chen K.H., Shao P.L., Sung P.H., Li Y.C., Chu Y.C., Guo J., Yip H.K. (2020). Uremic Toxic Substances are Essential Elements for Enhancing Carotid Artery Stenosis After Balloon-Induced Endothelial Denudation: Worsening Role of the Adventitial Layer. Am. J. Transl. Res..

[42] Chang M.C., Chang H.H., Chan C.P., Yeung S.Y., Hsien H.C., Lin B.R., Yeh C.Y., Tseng W.Y., Tseng S.K., Jeng J.H. (2014). p-Cresol Affects Reactive Oxygen Species Generation, Cell Cycle Arrest, Cytotoxicity and Inflammation/Atherosclerosis-Related Modulators Production in Endothelial Cells and Mononuclear Cells. PLoS ONE.

[43] Wong X., Carrasco-Pozo C., Escobar E., Navarrete P., Blachier F., Andriamihaja M., Lan A., Tomé D., Cires M.J., Pastene E. (2016). Deleterious Effect of P-Cresol on Human Colonic Epithelial Cells Prevented by Proanthocyanidin-Containing Polyphenol Extracts from Fruits and Proanthocyanidin Bacterial Metabolites. J. Agric. Food Chem..

[44] Idziak M., Pędzisz P., Burdzińska A., Gala K., Pączek L. (2014). Uremic Toxins Impair Human Bone Marrow-Derived Mesenchymal Stem Cells Functionality in Vitro. Exp. Toxicol. Pathol..

[45] Rong Y., Kiang T. (2021). Characterization of Human Sulfotransferases Catalyzing the Formation of p-Cresol Sulfate and Identification of Mefenamic Acid as a Potent Metabolism Inhibitor and Potential Therapeutic Agent for Detoxification. Toxicol. Appl. Pharmacol..

[46] James M.O., Ambadapadi S. (2013). Interactions of Cytosolic Sulfotransferases with Xenobiotics. Drug Metab. Rev..

[47] James M.O. (2014). Enzyme Kinetics of Conjugating Enzymes: PAPS Sulfotransferase. Methods Mol. Biol..

[48] Hart S.N., Li Y., Nakamoto K., Subileau E.A., Steen D., Zhong X.B. (2010). A Comparison of Whole Genome Gene Expression Profiles of HepaRG Cells and HepG2 Cells to Primary Human Hepatocytes and Human Liver Tissues. Drug Metab. Dispos..

[49] Bradshaw P.R., Athersuch T.J., Stachulski A.V., Wilson I.D. (2020). Acyl Glucuronide Reactivity in Perspective. Drug Discov. Today.

[50] Van Vleet T.R., Liu H., Lee A., Blomme E.A.G. (2017). Acyl Glucuronide Metabolites: Implications for Drug Safety Assessment. Toxicol. Lett..

[51] Tassaneeyakul W., Birkett D.J., Miners J.O. (1998). Inhibition of Human Hepatic Cytochrome P4502E1 by Azole Antifungals, CNS-Active Drugs and Non-Steroidal Anti-Inflammatory Agents. Xenobiotica.

[52] Karjalainen M.J., Neuvonen P.J., Backman J.T. (2008). In Vitro Inhibition of CYP1A2 by Model Inhibitors, Anti-Inflammatory Analgesics and Female Sex Steroids: Predictability of in Vivo Interactions. Basic Clin. Pharmacol. Toxicol..

