# Supplementary material for: Effects of p-Cresol on Oxidative Stress, Glutathione Depletion, and Necrosis in HepaRG Cells: Comparisons to Other Uremic Toxins and the Role of p-Cresol Glucuronide Formation
Source: Pharmaceutics. 2021 Jun 9;13(6):857. doi: 10.3390/pharmaceutics13060857 (PMC8228354; doi:10.3390/pharmaceutics13060857)
Supplement: Supplementary file 1 [file pharmaceutics-13-00857-s001.zip › pharmaceutics-1246505-supplementary.pdf]

pharmaceutics

MDPI

Supplementary Materials: Effects of *p*-Cresol on Oxidative Stress, Glutathione Depletion, and Necrosis in HepaRG Cells: Comparisons to Other Uremic Toxins and the Role of *p*-Cresol Glucuronide Formation

Sang Zhu, Yan Rong and Tony K. L. Kiang

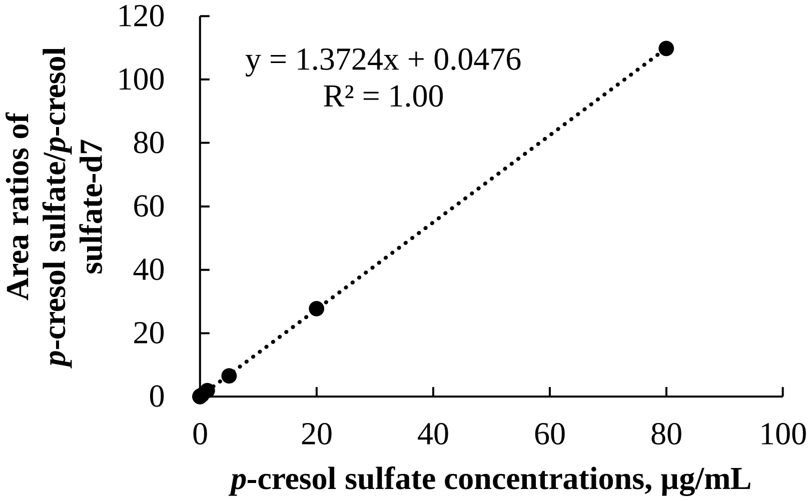

Figure S1. Calibration curve of *p*-cresol sulfate based on a weighted ($1/x^2$) least-squares regression model.

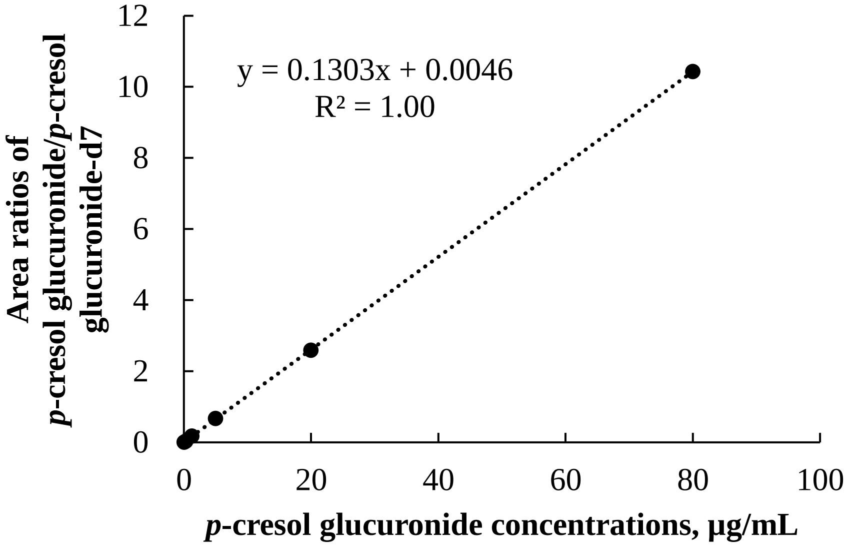

Figure S2. Calibration curve of *p*-cresol glucuronide based on a weighted ($1/x^2$) least-squares regression model.

Table S1. Accuracy and precision data of the UPLC/MS/MS assay for the measurement of *p*-cresol sulfate and *p*-cresol glucuronide.

	Nominal concentration	Intra-day 1, *n* = 5		Intra-day 2, *n* = 5		Intra-day 3, *n* = 5		Inter-day, *n* = 15	
		CV (%)	Accuracy (%)	CV (%)	Accuracy (%)	CV (%)	Accuracy (%)	CV (%)	Accuracy (%)
p-cresol sulfate	60 µg/mL (high QC)	4.31	102.88	5.12	95.39	7.78	98.09	6.84	106.07
	30 µg/mL (medium QC)	2.70	98.25	0.17	91.09	5.10	93.67	3.53	95.51
	0.004 ng/mL (low QC)	2.48	90.75	10.23	87.14	4.34	86.52	8.22	86.77
	0.001 ng/mL (LLOQ)	17.44	104.98	15.01	97.34	6.79	100.09	12.83	101.55
p-cresol glucuronide	60 µg/mL (high QC)	2.55	89.20	2.76	85.88	5.61	85.44	3.73	86.48
	30 µg/mL (medium QC)	3.30	95.79	6.89	92.69	5.12	88.56	4.22	92.27
	0.23 µg/mL (low QC)	7.61	113.92	1.03	111.47	0.09	108.19	2.35	111.69
	0.08 µg/mL (LLOQ)	3.47	107.59	0.87	105.21	1.41	102.04	1.53	103.94

CV, coefficient of variation; LLOQ, lower limit of quantification; QC, quality control; UPLC/MS/MS, ultra-high performance liquid chromatography-tandem mass spectrometry.

Table S2. Stability data of the UPLC/MS/MS assay for the measurement of *p*-cresol sulfate and *p*-cresol glucuronide.

Nominal Concentration	*p*-Cresol Sulfate		*p*-Cresol Glucuronide	
	0.004 ng/mL (Low QC)	60 µg/mL (High QC)	0.23 µg/mL (Low QC)	60 µg/mL (High QC)
	Accuracy (%)	Accuracy (%)	Accuracy (%)	Accuracy (%)
Autosampler stability	99.87	94.23	94.79	106.33
Bench-top stability	96.18	101.77	89.68	97.31
Freeze-thaw stability	113.25	91.01	92.79	104.12
Two-week stability	99.75	99.64	99.31	105.51

Various conditions were tested: 1) autosampler stability (i.e. 24 h at 4 °C), 2) bench-top stability (i.e. 6 h at "room temperature", 23.5 °C), 3) freeze-thaw stability (i.e. 3 cycles of freezing/thawing, where samples were frozen at −80 °C for 23.5 h then thawed at room temperature for 0.5 h), and 4) two-week stability (i.e. 2 weeks at −80 °C). *QC*, quality control; UPLC/MS/MS, ultra-high performance liquid chromatography-tandem mass spectrometry.

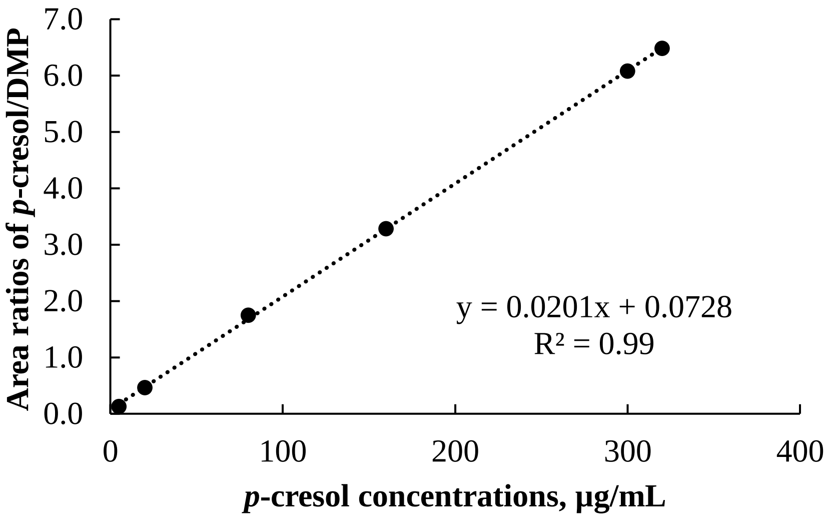

Figure S3. Calibration curve of *p*-cresol based on a weighted ($1/x^2$) least-squares regression model.

Table S3. Accuracy and precision data of the UPLC assay for the measurement of *p*-cresol.

Concentration	Intra-Day 1 (*n* = 5)		Intra-Day 2 (*n* = 5)		Intra-Day 3 (*n* = 5)		Intra-Day (*n* = 15)	
	CV (%)	Accuracy, %	CV (%)	Accuracy, %	CV (%)	Accuracy, %	CV (%)	Accuracy, %
300 µg/mL (high QC)	5.14	89.54	3.01	89.84	1.87	96.78	3.34	92.05
160 µg/mL (medium QC)	2.33	91.44	2.08	94.31	6.42	104.37	3.61	96.71
20 µg/mL (Low QC)	3.22	97.32	4.00	101.15	8.75	110.90	5.32	103.12
5 µg/mL (medium LLOQ)	6.03	102.11	5.07	102.46	8.01	98.77	6.37	101.11

CV, coefficient of variation; LLOQ, lower limit of quantification; QC, quality control; UPLC, ultra-high performance liquid chromatography.

Table S4. Stability data of the UPLC assay for the measurement of *p*-cresol.

Nominal Concentration (µg/mL)	20 µg/mL (Low QC)	300 µg/mL (High QC)
	Accuracy (%)	Accuracy (%)
Autosampler stability (%)	85.32	93.80
Bench-top stability (%)	104.93	113.01
Freeze-thaw stability (%)	105.60	113.79
Long-term stability (%)	101.32	99.66

Various conditions were tested: 1) autosampler stability (i.e. 24 h at 4 °C), 2) bench-top stability (i.e. 6 h at "room temperature", 23.5 °C), 3) freeze-thaw stability (i.e. 3 cycles of freezing/thawing, where samples were frozen at −80 °C for 23.5 h then thawed at room temperature for 0.5 h), and 4) long-term stability (i.e. 3 days at −80 °C). *QC*, quality control; UPLC, ultra-high performance liquid chromatography.
